# *Aedes aegypti* mosquito saliva ameliorates acetaminophen-induced liver injury in mice

**DOI:** 10.1371/journal.pone.0245788

**Published:** 2021-02-08

**Authors:** Josiane B. Assis, Bruno Cogliati, Eliane Esteves, Margareth L. Capurro, Denise M. Fonseca, Anderson Sá-Nunes

**Affiliations:** 1 Departamento de Imunologia, Instituto de Ciências Biomédicas, Universidade de São Paulo, São Paulo, Brazil; 2 Departamento de Patologia, Faculdade de Medicina Veterinária e Zootecnia, Universidade de São Paulo, São Paulo, Brazil; 3 Departamento de Parasitologia, Instituto de Ciências Biomédicas, Universidade de São Paulo, São Paulo, Brazil; 4 Instituto Nacional de Ciência e Tecnologia em Entomologia Molecular, Conselho Nacional de Desenvolvimento Científico e Tecnológico (INCT-EM/CNPq), Rio de Janeiro, Rio de Janeiro, Brazil; University of Louisville School of Medicine, UNITED STATES

## Abstract

Acetaminophen (*N*-acetyl-*p*-aminophenol, APAP) overdose is the most common cause of drug-induced liver injury (DILI). Although the primary hepatic damage is induced by APAP-derived toxic intermediates resulting from cytochrome P450 metabolism, immune components also play an important role in DILI pathophysiology. *Aedes aegypti* saliva is a source of bioactive molecules with *in vitro* anti-inflammatory and immunomodulatory activities. However, evidences on the therapeutic use of *Ae*. *aegypti* salivary preparations in animal models of relevant clinical conditions are still scarce. Thus, the present study was designed to evaluate the protective role of *Ae*. *aegypti* saliva in a murine model of APAP-induced DILI. C57BL/6 mice were exposed to *Ae*. *aegypti* bites 2 hours after APAP overdose. Biochemical and immunological parameters were evaluated in blood and liver samples at different time points after APAP administration. Exposure to *Ae*. *aegypti* saliva attenuated liver damage, as demonstrated by reduced hepatic necrosis and serum levels of alanine aminotransferase in APAP-overdosed mice. The levels of hepatic CYP2E1, the major enzyme responsible for the bioactivation of APAP, were not changed in *Ae*. *aegypti* exposed animals, suggesting no effects in the generation of hepatotoxic metabolites. On the other hand, mice treated with *Ae*. *aegypti* saliva following APAP overdose presented lower serum concentration of TNF-α, IL-6, IL-1β and IL-10, as well as reduced frequency of inflammatory cell populations in the liver, such as NKT cells, macrophages and dendritic cells. These findings show that *Ae*. *aegypti* saliva has bioactive molecules with therapeutic properties and may represent a prospective source of new compounds in the management of DILI-associated inflammatory disorders and, perhaps, many other inflammatory/autoimmune diseases.

## Introduction

Acetaminophen (*N*-acetyl-*p*-aminophenol, APAP), also known as paracetamol, is the main medication associated with drug-induced liver injury (DILI) and acute liver failure in the United States [1, 2]. APAP-related adverse events are still considered a public health burden, accounting for an approximate annual average of 112,000 calls to poison centers, 59,000 emergency department visits, and 38,000 hospitalizations, with an in-hospital mortality rate of 1.2% [2]. Despite being considered safe if used as recommended, APAP can cause severe hepatic injury when administered in high doses [3, 4].

At therapeutic doses, about 80–90% APAP is converted to sulphate and glucuronide conjugates, while 4–5% is excreted unchanged in the urine [5]. The residual APAP is metabolized by the cytochrome P450 (CYP450) system, especially by CYP2E1, the major enzyme responsible for the formation of N-acetyl-p-benzoquinone imine (NAPQI), a potent hepatotoxin [6, 7]. NAPQI reacts with glutathione, forming conjugates that are eliminated [8]. However, pathways of sulfate and glucuronic acid become saturated under overdose situations, shunting more APAP into the CYP450 system, increasing the amount of NAPQI and promoting a depletion of hepatic glutathione [9, 10]. As a consequence, multiple events including mitochondrial dysfunction and ATP depletion, DNA fragmentation, and modification of intracellular proteins contribute to the development of necrotic cell death [11].

Although toxic metabolites derived from APAP are responsible for primary hepatic injury, the immune system also plays an important role in liver failure. The recognition of damage-associated molecular patterns (DAMPs) released due to hepatocyte necrosis promotes inflammatory responses [12]. In addition, there is evidence that the severity of the hepatic lesion may depend on the subsequent involvement of inflammatory mediators and immune cells [13–15].

*Aedes aegypti* saliva is a source of bioactive molecules with anti-hemostatic, anti-inflammatory and immunomodulatory properties. The anti-hemostatic activities of *Ae*. *aegypti* saliva are well-characterized and include anticoagulant, antiplatelet and vasodilatory effects [16]. Regarding the anti-inflammatory and immunomodulatory roles, *in vitro* studies have shown that salivary preparations of *Ae*. *aegypti* are able to modulate the effector responses of immune cells such as mast cells [17], dendritic cells [18], macrophages [19, 20] and lymphocytes [21–24]. *In vivo* models revealed that the salivary secretion deposited in the host tissue during *Ae*. *aegypti* blood feeding creates a permissive environment that is appropriate for infectivity, replication and dissemination of a number of arboviruses transmitted by the mosquito, such as Zika, dengue, chikungunya, and yellow fever [25–29].

Despite the evident actions of *Ae*. *aegypti* saliva on the immune system, few studies have evaluated the therapeutic potential of the salivary complex [30, 31]. Therefore, in order to expand the spectrum of the therapeutic properties of *Ae*. *aegypti* salivary components to a condition of clinical importance, here we explored the immunomodulatory role of mosquito saliva on a murine model of APAP-induced liver injury.

## Material and methods

All the experiments involving mice were evaluated by the “Ethics Committee for Animal Use” from Instituto de Ciências Biomédicas, Universidade de São Paulo (ICB/USP–our Institutional Animal Care and Use Committee) and approved under the protocol numbers 16/2012 and 131/2017. The procedures are according to the Brazilian National Law number 11794 from 10/08/2008, which regulates all research activities involving animal use in the country. Anesthesia was performed for mosquito exposure (ketamine 50 mg/kg plus xylazine 20 mg/kg i.p.). Euthanasia was performed by exsanguination during sampling under halothane-induced anesthesia.

### Mice

Twelve- to twenty-week-old male C57BL/6 mice were acquired from the Faculdade de Medicina, Universidade de São Paulo (FMUSP) and maintained in the animal facility of the Departamento de Imunologia from ICB/USP. For the experiments, animals were kept with water and food *ad libitum*, except when indicated.

### Mosquitoes

Pathogen-free *Ae*. *aegypti* mosquitoes were bred in an insectary at Departamento de Parasitologia from ICB/USP. The mosquitoes were separated into groups of 30 females and kept in plastic containers covered with tulle fabric. A cotton cloth soaked with 10% sucrose solution (w/v) was placed on the fabric to keep the mosquitoes hydrated and fed, and it was removed 12 hours before mice exposure to the mosquitoes.

### APAP-induced liver injury

The murine model of APAP-induced DILI was adapted from a previously described methodology [32]. Briefly, mice were starved for 15–16 hours before APAP administration. APAP was diluted in PBS, heated to 60°C until complete solubilization and slowly cooled to 30–37°C for administration. Mice were intraperitoneally injected with the APAP solution (300 mg/kg body weight) or PBS and regained free access to food.

### Mice exposure to *Ae*. *aegypti* bites

To evaluate the effects of *Ae*. *aegypti* saliva on the mice model of APAP-induced liver injury, we have adapted a previously published protocol of mosquito exposure [33]. Briefly, animals were anesthetized with a subcutaneous injection of ketamine and xylazine (50 mg/kg and 20 mg/kg, respectively), diluted in physiological saline, two hours after APAP administration. One-half of APAP-injected mice was kept in the cage (“APAP” group) while the other half was placed on the top of the plastic containers with the mosquitoes for 30 minutes (“APAP+Bites” group). Negative control groups consisted in animals inoculated with PBS and not exposed to mosquito bites (“PBS” group) and animals inoculated with PBS and exposed to the bites of 30 mosquitoes (“PBS+Bites” group). Mice were euthanized 6, 12 and 24 hours after APAP or PBS injection by exsanguination during sampling under halothane-induced anesthesia.

### Quantification of serum aminotransferases

Blood, collected from inferior vena cava 6, 12 and 24 hours after injection, was centrifuged (10 minutes, 1500 × *g*), and serum was stored at -20°C. The serum levels of alanine aminotransferase (ALT) and aspartate aminotransferase (AST) were measured with an automated spectrophotometric analyzer (Labmax 240, Labtest Diagnostica, Lagoa Santa, MG, Brazil). Values were expressed in IU/L.

### Liver histopathology

Livers were excised and fragments were fixed in 10% phosphate-buffered formalin (Synth, Diadema, SP, Brazil) for up to 48 hours, transferred to 70% ethanol, then to xylene, and embedded in paraffin. Tissue transversal sections of 5 μm was stained with hematoxylin and eosin and the slides were analyzed by light microscopy (Leica Microsystems, Wetzlar, Germany). Analysis of liver necrosis area in APAP-injected animals exposed or not to mosquitoes was performed using ImageJ program (National Institutes of Health, Bethesda, MD, USA) in 10 randomly selected fields (10× objective) per section from each liver. Data analysis was based on the following formula: necrosis area (%) = (necrosis area ×100)/(total field area—vascular luminal area).

### Western blot

Flash frozen liver tissue was homogenized in RIPA buffer (150 mM NaCl, 1% NP40, 0.1% SDS, 50 mM Tris; pH 8.0) containing 1% protease inhibitor (Sigma-Aldrich) and centrifuged at 20,000 × *g* for 10 minutes at 4°C. The protein concentration was determined using the BCA Protein Assay Kit (Thermo Fisher Scientific) according to the manufacturer’s instructions. Proteins were separated on acrylamide gels using electrophoresis and blotted onto nitrocellulose membranes as described [20]. Membranes were blocked with TBS-T buffer (Tris-buffered saline, 0.1% Tween-20) containing 10% fetal bovine serum (FBS) for 1 hour. Membranes were incubated for 1 hour with rabbit anti-mouse CYP2E1 polyclonal antibody (Biomatik, Cambridge, ON, CA, USA) followed by incubation for 1 hour with anti-rabbit antibodies conjugated with horseradish peroxidase (Cell Signaling Technology, Danvers, MA, USA). Antibody excess was removed by washing the membranes several times in TBS-T. CYP2E1 protein was detected by chemiluminescent ECL Detection Kit (Thermo Fisher Scientific) in a photodocumentation system (G:BOX, Syngene, Cambridge, UK). Membranes were stripped with Restore^TM^ Stripping Buffer (Thermo Fisher Scientific) for 15 minutes, washed, blocked again and incubated overnight at 4°C with rabbit anti-mouse β-actin antibody (Cell Signaling Technology). Membranes were washed and incubated for 1 hour with anti-rabbit horseradish peroxidase-conjugated antibody (Cell Signaling Technology), and β-actin bands were visualized as described earlier. The density of the bands was analyzed using Digi-Doc1000 software (Alpha Innotech Corporation, San Leandro, CA, USA) and the values normalized by the total of β-actin present in each sample and presented as percentage of protein in relation to the control (PBS) group.

### Cytokine quantification

The determination of serum TNF-α, IL-1β, IL-6 and IL-10 was performed by OptEIA™ ELISA sets (BD Biosciences, San Diego, CA, USA), according to the manufacturer’s recommendations. Values were expressed as pg/mL deduced from standard curves of recombinant cytokines ran in parallel. The detection limit for each cytokine analyzed was 15.6 pg/mL (TNF-α and IL-6) and 31.3 pg/mL (IL-1β and IL-10).

### Flow cytometry

Liver samples, collected 24 hours after APAP or PBS injection, were dispersed in small fragments, digested in 3 mL of RMPI 1640 medium (Thermo Fisher Scientific, Waltham, MA, USA) containing 0.5 mg/mL collagenase IV (Sigma-Aldrich) and 0.2 mg/mL DNAse I (Sigma-Aldrich, St. Louis, MO, USA) for 20 minutes at 37°C under 90 rpm stirring. The digestion was blocked with 10 mL of PBS containing 10% of inactivated FBS, the fragments were pressed through a 40 μm cell strainer (Corning, Durhan, NC, USA) with a syringe plunger and centrifuged for 3 minutes (20 × *g*, 4°C). The supernatants were collected and centrifuged again for 5 minutes (300 × *g*, 4°C), the cell pellets were resuspended in 13 mL of Percoll 35% (Sigma-Aldrich) and then centrifuged for 25 minutes (850 × *g*, 20°C) without brake for cell separation. After centrifugation, the cell-free supernatants were discarded and the erythrocytes were lysed with ACK buffer (Thermo Fisher Scientific), followed by the addition of PBS containing 2% FBS to stop the reaction. After further centrifugation for 5 minutes (300 × *g*, 4°C), the cell pellets were resuspended in PBS containing 1% FBS. Cells resulting from this separation process were counted, stained with fluorochrome-conjugated monoclonal antibodies, and then immunophenotyped by flow cytometry (FACSCanto II, BD Bioscience). The S1 Fig. presents the gating strategy used to analyze major cell populations in the liver. Briefly, cells were first gated for *singlets* (FSC-H *versus* FSC-A) followed by positive events for the CD45 marker (a membrane glycoprotein that characterizes cells of hematopoietic origin) and negative events for LIVE/DEAD viability marker (Thermo Fisher Scientific), being considered as “immune cells”. Lymphoid cells were identified according to the following markers: CD3^+^CD4^+^CD8^-^ (CD4^+^ T cells), CD3^+^CD4^-^CD8^+^ (CD8^+^ T cells), CD3^-^CD19^+^ (B cells), CD3^-^CD19^-^NK1.1^+^ (NK cells), CD3^+^CD19^-^NK1.1^+^ (NKT cells). Myeloid cells were identified according to the following markers: CD11b^+^F4/80^+^ (macrophages) and CD11b^+^F4/80^-^CD11c^+^MHC II^+^ (dendritic cells).

### Statistical analysis

Statistical analyses of differences between the means of experimental groups were performed using analysis of variance (ANOVA) followed by Tukey as a post-test. A value of *p* ≤ 0.05 was considered statistically significant.

## Results

### Exposure to *Ae*. *aegypti* bites ameliorates APAP-induced liver injury

**Fig 1:**
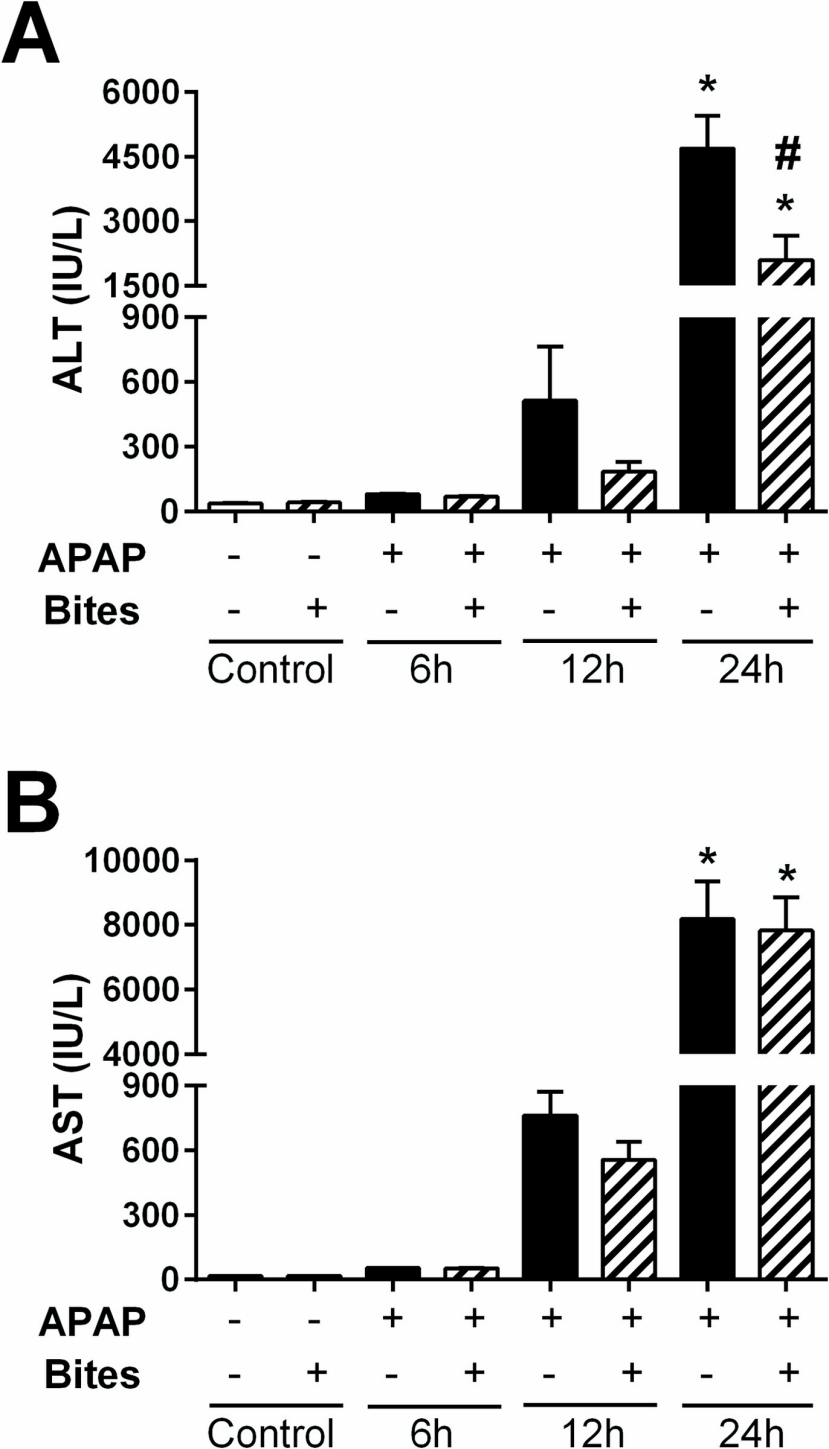
Serum levels of ALT and AST in mice injected with APAP and exposed to *Ae*. *aegypti* mosquito bites. C57BL/6 male mice were injected with PBS or APAP (300 mg/kg i.p.) and, after 2 hours, exposed to 30 *Ae*. *aegypti* mosquitoes. Transaminase levels were measured in samples collected 6, 12 and 24 hours after PBS or APAP injection. **A.** ALT levels; **B.** AST levels. The data represent the mean ± SEM (*n* = 4–12). * *p* < 0.05 *versus* “PBS” group; ^**#**^ *p* < 0.05 *versus* “APAP” group at 24 h after injection.

In order to evaluate the therapeutic effects of *Ae*. *aegypti* saliva in the DILI, mice were exposed to mosquito bites 2 hours after APAP overdose and had the serum levels of ALT and AST measured at different time points. Although an increase in the levels of both liver transaminases was detected at 6 and 12 hours after APAP injection, only at 24 hours the levels were statistically significant when compared to the control groups (“PBS” and “PBS+Bites”). At this time point, the exposure of APAP-overdosed mice to mosquito bites prevented ALT increase by 55% compared to the non-exposed APAP group (Fig 1A). No differences were noted regarding AST values between “APAP” and “APAP+Bites” groups at all time points evaluated (Fig 1B).

**Fig 2:**
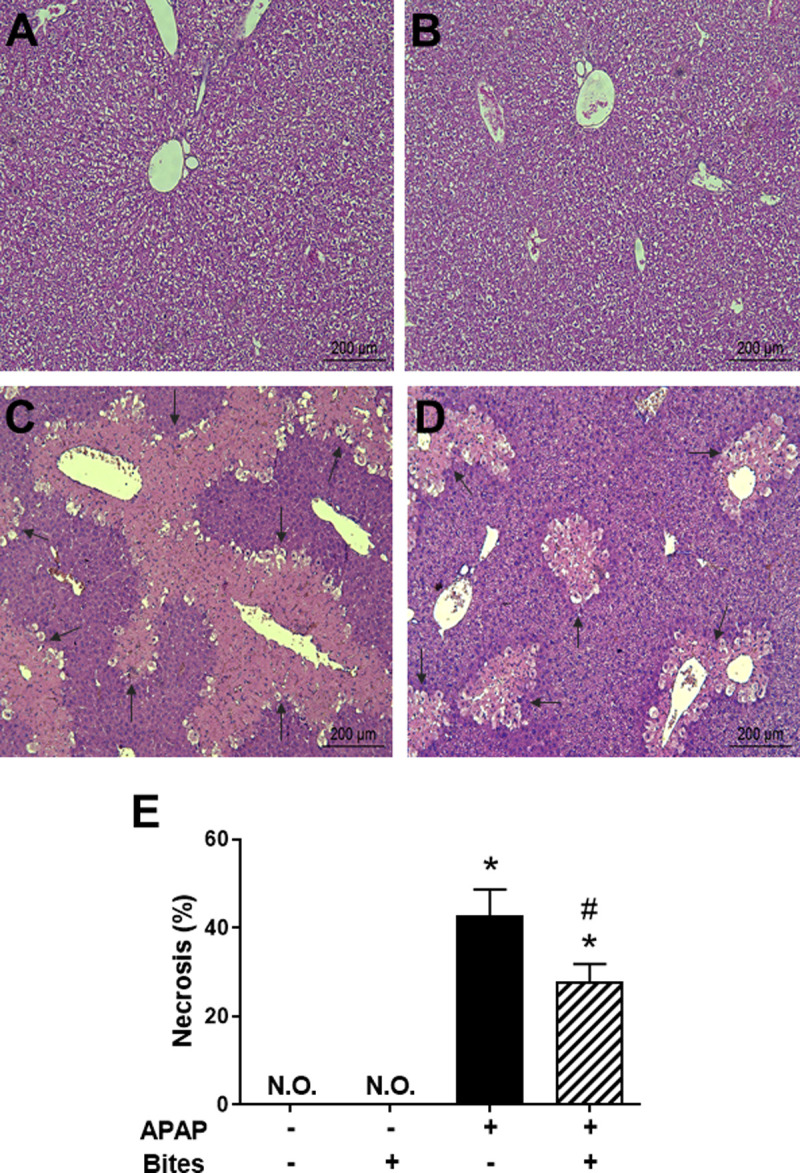
Liver histopathology of mice injected with APAP and exposed to *Ae*. *aegypti* bites. C57BL/6 male mice were injected with PBS or APAP (300 mg/kg i.p.) and, after 2 hours, exposed to 30 *Ae*. *aegypti* mosquitoes. Liver samples collected 24 hours after injection were assessed for the extent of damage caused by APAP overdose. **A.** “PBS” group; **B.** “PBS+Bites” group; **C.** “APAP” group; **D.** “APAP+Bites” group; **E.** Percentual of necrotic areas in the livers. Sections were analyzed at 100 × magnification. The arrows indicate necrotic areas. The data in **E** represent the mean ± SEM (*n* = 6). * *p* < 0.05 *versus* “PBS” group; ^**#**^ *p* < 0.05 *versus* “APAP” group. N.O.: not observed.

Considering the 130-fold increase in basal ALT levels and the 450-fold increase in basal AST levels after 24 hours of APAP inoculation, histological analysis of liver samples was performed at this time point. The liver histology of mice injected with PBS showed the normal architecture of the organ, with lobule units containing portal tracts along the periphery, a central vein and preserved hepatocytes arranged in thin cords (Fig 2A). The exposure to mosquito bites did not change the normal architecture of the organ and no signs of cell damage or inflammation were observed (Fig 2B). As expected, the liver of APAP-overdosed mice presented intense areas of necrosis and inflammatory infiltrate (Fig 2C). Nonetheless, when exposed to *Ae*. *aegypti* mosquito bites, APAP-injected mice clearly presented less inflammation and self-limited areas of necrotic lesions (Fig 2D). Accordingly, the morphometric analysis confirmed that *Ae*. *aegypti* saliva reduced the liver necrosis induced by APAP (Fig 2E). Taken all together, these findings demonstrate the ability of *Ae*. *aegypti* saliva to reduce liver damage resulting from APAP overdose.

### *Aedes aegypti* saliva does not change the levels of hepatic CYP2E1

**Fig 3:**
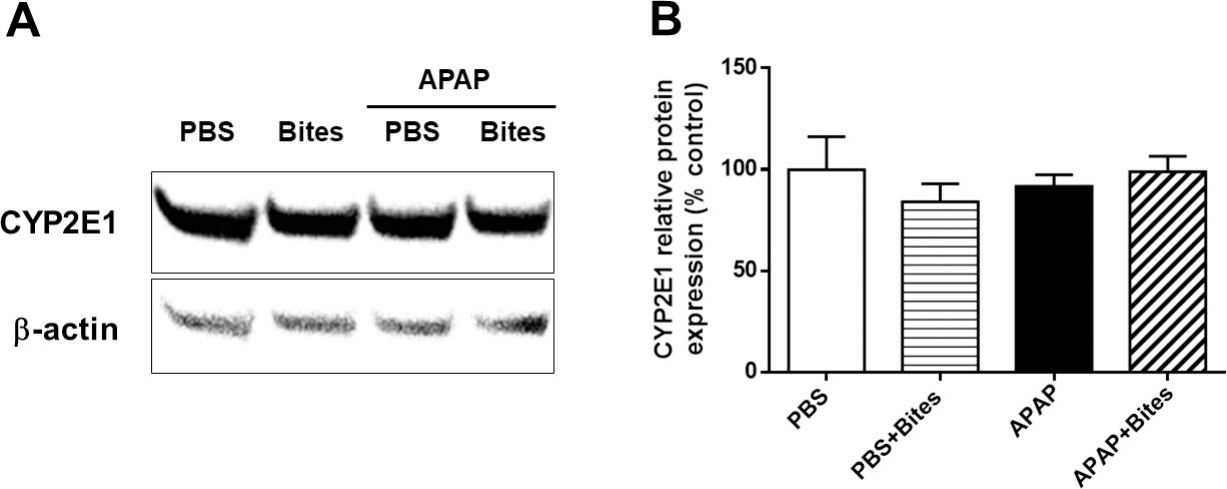
Expression of hepatic CYP2E1 in mice injected with APAP and exposed to *Ae*. *aegypti* mosquito bites. C57BL/6 male mice were injected with PBS or APAP (300 mg/kg i.p.) and, after 2 hours, exposed to 30 *Ae*. *aegypti* mosquitoes. Liver samples collected 6 hours after injection were processed for evaluation of CYP2E1 expression. **A.** Representative CYP2E1 protein bands and respective β-actin bands evaluated by Western blot; **B.** Relative expression of CYP2E1 was determined by densitometry. The data represent the mean ± SEM (*n* = 4).

In order to evaluate whether *Ae*. *aegypti* saliva is influencing the conversion of APAP to NAPQI, hepatic CYP2E1 was measured. Immunoblot analysis revealed that CYP2E1 protein levels were not significantly different among the experimental groups (Fig 3). These findings suggest that *Ae*. *aegypti* saliva does not influence APAP metabolism via CYP450 system.

### *Ae*. *aegypti* saliva downmodulates the production of serum cytokines and liver infiltration of inflammatory cells in APAP-overdosed mice

**Fig 4:**
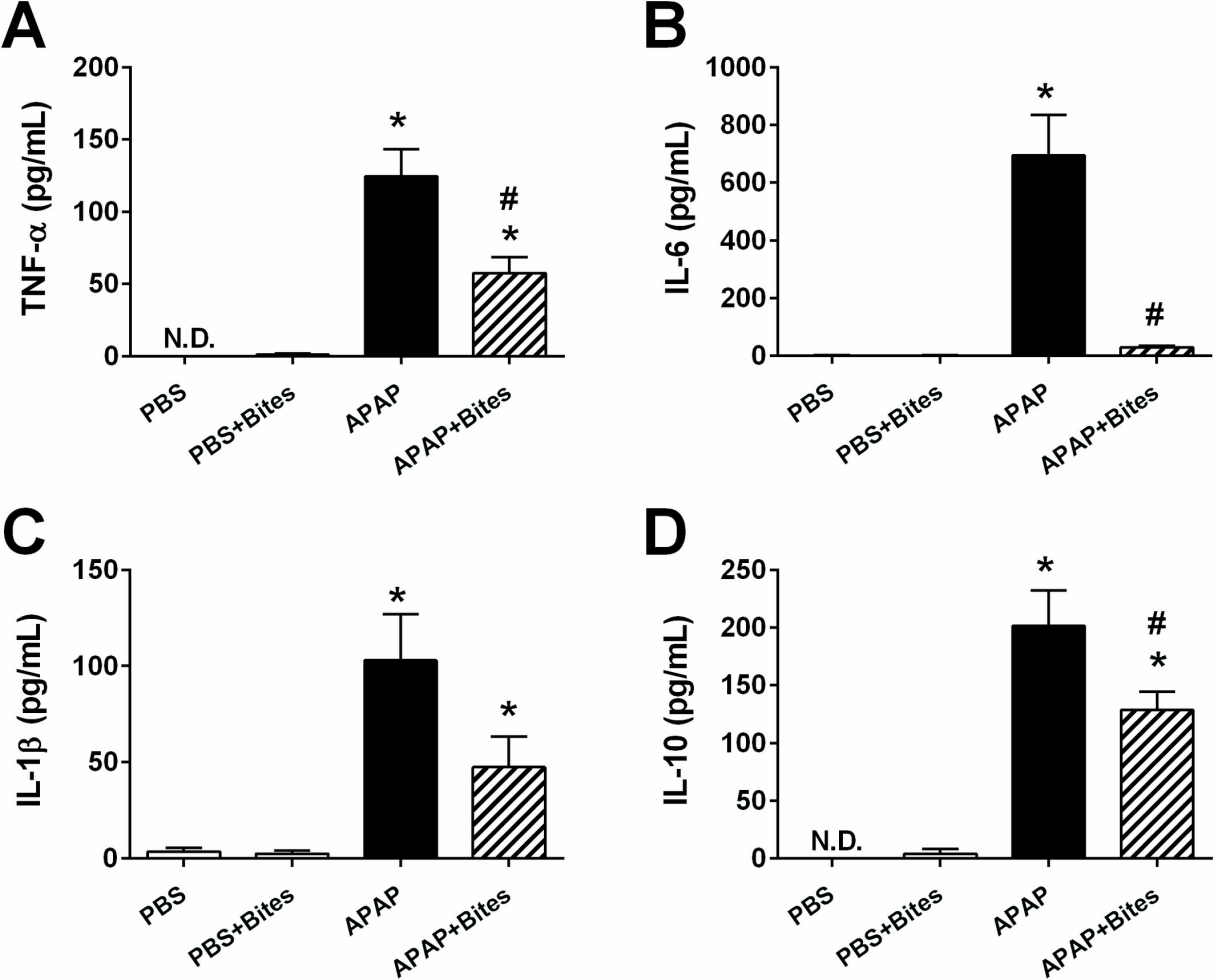
Cytokine production in the serum of mice injected with APAP and exposed to *Ae*. *aegypti* bites. C57BL/6 male mice were injected with PBS or APAP (300 mg/kg i.p.) and, after 2 hours, exposed to 30 *Ae*. *aegypti* mosquitoes. Blood was collected 24 hours after inoculation with PBS or APAP and cytokines were evaluated in the serum by ELISA. **A.** TNF-α; **B.** IL-6; **C.** IL-1β; and **D.** IL-10. The data represent the mean ± SEM (*n* = 5–10). * *p* < 0.05 *versus* “PBS” group; ^**#**^ *p* < 0.05 *versus* “APAP” group. N.D.: not detected.

Since exposure to mosquito bites did not influence the formation of hepatotoxic metabolites and, considering the literature data about the immunomodulatory activity of *Ae*. *aegypti* saliva, we proceeded to assess the influence of saliva on the immune response in APAP-induced hepatotoxicity. Almost undetectable serum concentrations of TNF-α, IL-6, IL-1β and IL-10 were observed in mice from “PBS” and “PBS+Bites” groups while APAP inoculation increased the concentration of all these cytokines. Mice exposed to *Ae*. *aegypti* bites following APAP injection produced significantly less TNF-α (Fig 4A), IL-6 (Fig 4B) and IL-10 (Fig 4D). Although the reduction of serum IL-1β in “APAP+Bites” group did not reach statistical significance when compared to the “APAP” group, it was reduced by more than 50% (Fig 4C).

**Fig 5:**
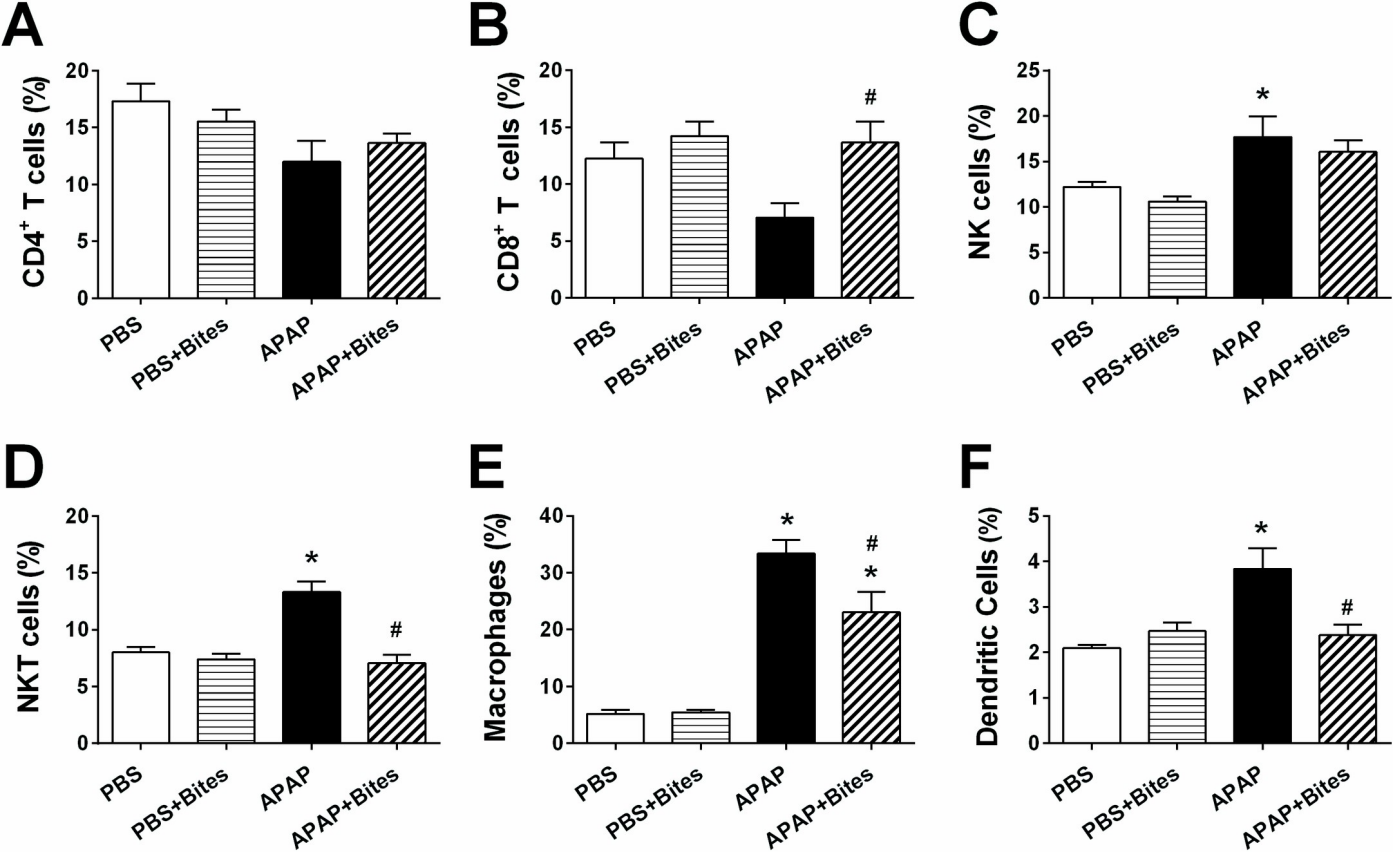
Liver immunophenotyping in mice injected with APAP and exposed to *Ae*. *aegypti* bites. C57BL/6 male mice were injected with PBS or APAP (300 mg/kg i.p.) and, after 2 hours, exposed to 30 *Ae*. *aegypti* mosquitoes. Liver samples collected 24 hours after injection were processed and immunophenotyping of hepatic cells was performed by flow cytometry. **A.** CD4^+^ T cells; **B.** CD8^+^ T cells; **C.** NK cells; **D.** NKT cells; **E.** Macrophages; and **F.** Dendritic cells. The data represent the mean ± SEM (*n* = 4–14). * *p* < 0.05 *versus* “PBS” group; ^**#**^ *p* < 0.05 *versus* “APAP” group.

Next, the frequency of major leukocyte populations in the liver of mice was determined by flow cytometry and compared among the experimental groups. The exposure to mosquito bites in PBS injected animals did not significantly change the percentage of any of the cell populations evaluated when compared to mice not exposed to the mosquitoes. The inoculation of APAP produced no significant changes in the percentage of B cells (data not shown), CD4^+^ T cells (Fig 5A) or CD8^+^ T cells (Fig 5B). On the other hand, APAP injection induced an increase of NK cells (Fig 5C), NKT cells (Fig 5D), macrophages (Fig 5E) and dendritic cells (Fig 5F). Strikingly, the exposure of APAP-inoculated mice to mosquito saliva partially prevented the increase of macrophages (Fig 5E), while the frequency of NKT cells (Fig 5D) and dendritic cells (Fig 5F) was reduced to levels similar to those found in “PBS” and “PBS+Bites” groups.

S2 Fig presents the comparative proportion of all immune cell populations for each experimental condition. In the liver of the “PBS” group, there was a predominance of lymphoid populations (B cells, T cells, NK cells and NKT cells) while myeloid populations (macrophages and dendritic cells) represented a smaller fraction of the total cells. A similar profile was observed for the “PBS+Bites” group. The inoculation of APAP increased both myeloid populations evaluated and only the NK and NKT cells in lymphoid populations, while the remaining populations were proportionally decreased. The exposure of APAP-overdosed mice to mosquito saliva modulated the cell distribution to an intermediate profile, with the proportion of some cell populations being similar to those found in the “PBS” and “PBS+Bites” groups (CD8^+^ T cells, NKT cells and dendritic cells) while other cell populations remained similar to the “APAP” group (CD4^+^ T cells, B cells and NK cells).

## Discussion

As a consequence of their mechanisms of action, saliva and salivary molecules originated from hematophagous arthropods have been prospected for prevention or treatment of clinical conditions [29, 34–39]. Curiously, despite the direct identification of over 1,200 proteins in the salivary glands of *Ae aegypti* by LC-MS/MS analysis [40], the species’ saliva remains little explored in animal models of diseases when compared with other arthropod groups [41, 42]. These restricted findings prompted us to expand the evaluation of *Ae*. *aegypti* saliva in a murine model of pharmacologically-induced acute liver failure.

The APAP dose employed in our study evidenced the toxic action of the drug to the liver, generating hepatocellular death and increased ALT and AST levels associated with an inflammatory process, thus reproducing important parameters of DILI and acute liver failure in humans [43]. The considerable reduction in liver necrosis and serum ALT levels, which is a more specific biochemical marker for assessing liver damage than AST [44], point to a hepatoprotective role of *Ae*. *aegypti* saliva against APAP intoxication.

The primary liver damage caused by APAP overdose is due to the toxicity of the NAPQI metabolite, generated after APAP metabolism mostly by CYP2E1 [6], although a minor role of other isoforms is reported [7]. A direct influence of *Ae*. *aegypti* saliva in this pathway was ruled out, since no difference in CYP2E1 protein levels was found. Similar hepatoprotective effects without changes in CYP2E1 expression were previously reported by others [45, 46]. These results reinforce the assumption of mosquito saliva having a preventive action on immune-mediated liver damage secondary to APAP overdose.

Previous studies have investigated the involvement of the inflammatory response in APAP-induced liver injury through the evaluation of cellular and humoral components. Blazka *et al*. demonstrated that the proinflammatory cytokines TNF-α and IL-1α are released in response to the drug intoxication [47]. Our study confirmed that APAP administration increased TNF-α and IL-1β, in addition to IL-6, with evident decrease of these levels in the animals exposed to mosquito saliva. Accordingly, *in vitro* studies showed the inhibition of proinflammatory cytokines by *Ae*. *aegypti* salivary gland extract (SGE) [18, 20]. Although unexpected, the higher levels of IL-10 in APAP-treated animals was demonstrated previously [32] and may represent attempt of the organism to regulate the inflammatory reaction resulting from APAP administration.

The liver has constitutive resident immune cells such as Kupffer cells (hepatic macrophages), dendritic cells, NK cells and NKT cells. Such cells seem to play an important role both in liver homeostasis and in the immunopathology of DILI, autoimmune and viral hepatitis in humans [48]. In our study, hepatic macrophages were also increased in the “APAP” group. As revealed in animal models and in corresponding translational studies of patients with acute liver failure, necrotic hepatocytes release DAMPs, which are recognized by Kupffer cells, leading to their activation [15]. Activated hepatic macrophages release several proinflammatory cytokines, such as IL-1β and IL-6, which are directly related to the promotion of the inflammatory process [49], as well as TNF-α, which is highly relevant for sensitization of hepatocytes for apoptosis [50]. We demonstrated that mosquito saliva partially inhibited the increase of hepatic macrophages observed in APAP-overdosed mice. Recently, we have showed that *Ae*. *aegypti* SGE negatively interferes with the macrophage polarization to an inflammatory (M1) profile *in vitro* by reducing the production of nitric oxide, IL-6 and IL-12 while increasing the production of IL-10 [20]. However, given the complexity of the *in vivo* model evaluated, it is not possible to assume that the decrease of the serum cytokines in the “APAP+Bites” group is a direct consequence of the macrophage reduction and/or function in the liver.

The increase of NK, NKT and dendritic cells was also observed in APAP-injected mice. Such findings contradict some studies on the role of these cells in the hepatic injury induced by APAP. It has been shown that NKT-deficient mice (CD1d^-/-^ and Jα18^-/-^ mice) are more susceptible to fulminant hepatic failure due an marked production of ketone bodies and increased levels of CYP2E1 [51]. In contrast, another study using Jα18^-/-^ mice (which are selectively deficient in Vα14*i* NKT cells) showed attenuated APAP hepatotoxicity due to increased hepatic glutathione levels that, in turn, detoxifies the metabolites of APAP and suppress liver damage [52]. Still, C57BL/6 mice depleted of NK and NKT cells had less liver damage and longer survival than control animals when injected with high doses of APAP [13]. Regarding dendritic cells, a dual role has been reported upon APAP challenge. On one hand, liver dendritic cells expressed higher levels of MHC class II, costimulatory molecules, innate recognition recetors and inflammatory cytokines after APAP injection. On the other hand, depletion of dendritic cells increased APAP-mediated toxicity and mortality. It has been suggested that dendritic cells protective role was due to a downregulation of NK cell activation [53]. As far as we know, the direct effect of *Ae*. *aegypti* salivary preparations on NK and/or NKT cells was never reported. However, our group demonstrated that the mosquito SGE was not able to affect the differentiation, maturation or function of murine dendritic cells *in vitro* [24]. The fact that mosquito saliva decreased the percentage of dendritic cells in APAP-injected mice suggests that salivary components might act in a different way *in vivo*.

Recent studies evaluating the anti-inflammatory/immunomodulatory activities of *Ae*. *aegypti* saliva have uncovered a rich source of bioactive components with therapeutic potential. In a murine model of sepsis, pretreatment with *Ae*. *aegypti* SGE reduced the mortality of mice, the bacterial load and neutrophil infiltration to the peritoneal cavity while increasing nitric oxide production and antioxidant defenses [30]. *Ae*. *aegypti* SGE also ameliorated the symptoms of experimental colitis by improving clinical and postmortem scores and diminishing the inflammatory areas and the production of inflammatory cytokines in the colon [31]. While *in vitro* studies have shown that salivary preparations of *Ae*. *aegypti* are able to modulate the effector responses of immune cells [17–24], the glimpses into the mechanisms of action of some salivary molecules could explain the phenotype observed in our assays. For example, *Ae*. *aegypti* saliva contains an apyrase [54] and adenosine deaminase [55] which hydrolyzes their substrates to adenosine monophosphate and inosine, respectively, and both products are potent inhibitors of inflammatory cytokines. The salivary members of the D7 family, AeD7L1 and AeD7L2, bind lipid mediators involved in proinflammatory responses [56, 57]. A 34-kDa salivary protein identified in salivary *Ae*. *aegypti* salivary glands inhibits the mRNA expression of type I IFN and IFN-regulatory factors [58]. LTRIN downregulates the activation of NF-κB and the production of proinflammatory cytokines by binding the lymphotoxin-β receptor in many cell types [59]. Whether one or more of these salivary molecules account for the protective effects on APAP overdose evidenced in our study, it remains to be elucited.

## Conclusion

*Ae*. *aegypti* saliva ameliorates APAP-induced liver injury by preventing the amplification of immune-mediated liver necrosis. The protective effect observed is associated with downmodulation of proinflamatory cytokines and immune cells involved in the acute liver disease, but with no apparent changes in APAP metabolism by CYP2E1. Future studies employing fractionation techniques may help to identify and characterize putative molecule(s) involved in this modulation. Our study reinforces the potential of salivary components of *Ae*. *aegypti* to be employed in the prevention and/or treatment of DILI and other inflammatory clinical conditions.

## Supporting information

S1 Fig Gating strategy for the flow cytometric analysis of myeloid and lymphoid populations in the liver. Cells were prepared as described in Materials and Methods and gated for *singlets* (FSC-H *vs*. FSC-A), followed by positive events for the CD45 marker (a membrane glycoprotein that characterizes cells of hematopoietic origin) and negative events for LIVE/DEAD viability marker. Lymphoid cells were identified according to the following markers: CD3^+^CD4^+^CD8^-^ (CD4^+^ T cells), CD3^+^CD4^-^CD8^+^ (CD8^+^ T cells), CD3^-^CD19^+^ (B cells), CD3^-^CD19^-^NK1.1^+^ (NK cells), CD3^+^CD19^-^NK1.1^+^ (NKT cells). Myeloid cells were identified according to the following markers: CD11b^+^F4/80^+^ (macrophages) and CD11b^+^F4/80^-^CD11c^+^MHC II^+^ (dendritic cells).
(TIF)

S2 Fig Relative percentage of each leukocyte population in the liver of mice injected with APAP and exposed to *Ae*. *aegypti* saliva. The percentage (%) presented represents each cell type in relation to the total leukocytes (CD45^+^ cells) in each group.
(TIF)
