# Supplementary figures and images for: Aedes aegypti mosquito saliva ameliorates acetaminophen-induced liver injury in mice
Source: PLoS One. 2021 Feb 8;16(2):e0245788. doi: 10.1371/journal.pone.0245788 (PMC7869984; doi:10.1371/journal.pone.0245788)

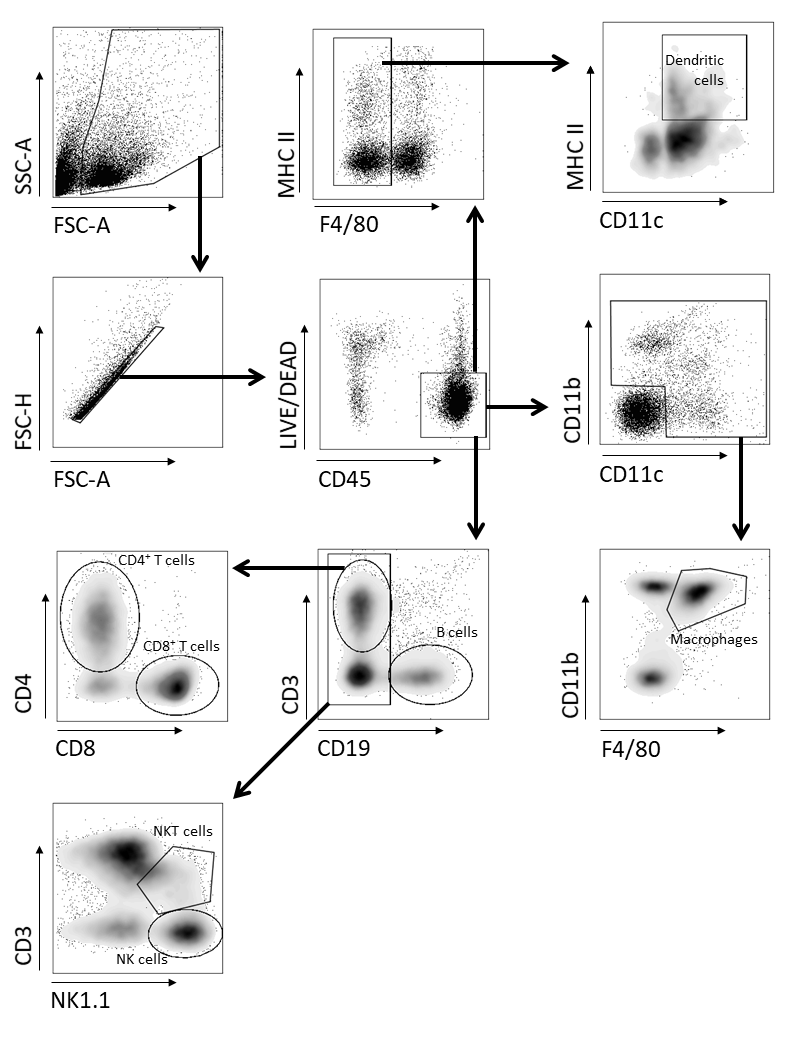

Supplement: S1 Fig — Cells were prepared as described in Materials and Methods and gated for singlets (FSC-H vs. FSC-A), followed by positive events for the CD45 marker (a membrane glycoprotein that characterizes cells of hematopoietic origin) and negative events for LIVE/DEAD viability marker. Lymphoid cells were identified according to the following markers: CD3+CD4+CD8- (CD4+ T cells), CD3+CD4-CD8+ (CD8+ T cells), CD3-CD19+ (B cells), CD3-CD19-NK1.1+ (NK cells), CD3+CD19-NK1.1+ (NKT cells). Myeloid cells were identified according to the following markers: CD11b+F4/80+ (macrophages) and CD11b+F4/80-CD11c+MHC II+ (dendritic cells). (TIF) [file pone.0245788.s001.tif]

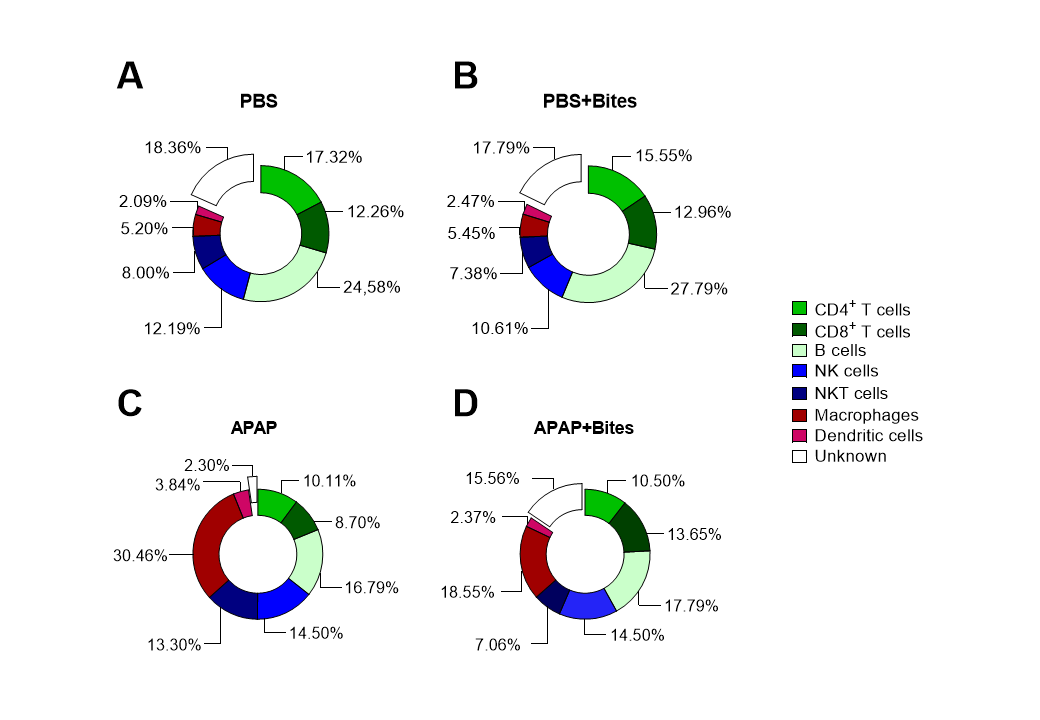

Supplement: S2 Fig — The percentage (%) presented represents each cell type in relation to the total leukocytes (CD45+ cells) in each group. (TIF) [file pone.0245788.s002.tif]
